# Broadband Photon Harvesting in Organic Photovoltaic Devices Induced by Large-Area Nanogrooved Templates

**DOI:** 10.1021/acsanm.3c00553

**Published:** 2023-04-03

**Authors:** Debasree Chowdhury, Shaimaa A. Mohamed, Giacomo Manzato, Beatrice Siri, Roberto Chittofrati, Maria Caterina Giordano, Mohamed Hussein, Mohamed F. O. Hameed, Salah S. A. Obayya, Philipp Stadler, Markus C. Scharber, Giuseppe Della Valle, Francesco Buatier de Mongeot

**Affiliations:** † Department of Physics, University of Genova, Via Dodecaneso 33, 16146 Genova, Italy; ‡ Centre for Photonics and Smart Materials, Zewail City of Science, Technology and Innovation, October Gardens, 6th of October City, Giza 12578, Egypt; § Centre for Nanotechnology, Zewail City of Science, Technology and Innovation, October Gardens, 6th of October City, Giza 12578, Egypt; ∥ Nanotechnology and Nanoelectronics Engineering Program, Zewail City of Science, Technology and Innovation, October Gardens, 6th of October City, Giza 12578, Egypt; ⊥ Physical Chemistry, Linz Institute for Organic Solar Cell (LIOS), Johannes Kepler University Linz, Altenbergerstr, 69, A-4040 Linz, Austria; # Department of Physics, Faculty of Science, Ain Shams University, Abbassia, 11566 Cairo, Egypt; ∇ Light Technology Institute, Karlsruhe Institute of Technology, Engesserstrasse 13, 76131 Karlsruhe, Germany; ○ Mathematics and Engineering Physics Department, Faculty of Engineering, University of Mansoura, Mansoura 35516, Egypt; ◆ Department of Electronics and Communication Engineering, Faculty of Engineering, University of Mansoura, Mansoura 35516, Egypt; ¶ Dipartimento di Fisica and IFN-CNR, Politecnico di Milano, Piazza Leonardo da Vinci, 32-20133 Milano, Italy

**Keywords:** nanofabrication, light trapping, thin-film photovoltaics, broadband, nanograting silica, organic semiconductor

## Abstract

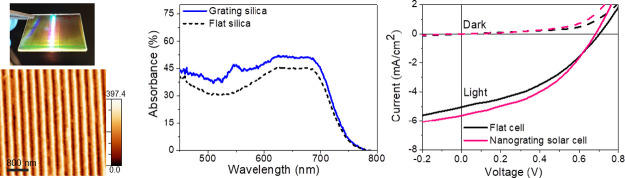

Thin-film organic photovoltaic (OPV) devices represent an attractive alternative to conventional silicon solar cells due to their lightweight, flexibility, and low cost. However, the relatively low optical absorption of the OPV active layers still represents an open issue in view of efficient devices that cannot be addressed by adopting conventional light coupling strategies derived from thick PV absorbers. The light coupling to thin-film solar cells can be boosted by nanostructuring the device interfaces at the subwavelength scale. Here, we demonstrate broadband and omnidirectional photon harvesting in thin-film OPV devices enabled by highly ordered one-dimensional (1D) arrays of nanogrooves. Laser interference lithography, in combination with reactive ion etching (RIE), provides the controlled tailoring of the height and periodicity of the silica grooves, enabling effective tuning of the anti-reflection properties in the active organic layer (PTB7:PCBM). With this strategy, we demonstrate a strong enhancement of the optical absorption, as high as 19% with respect to a flat device, over a broadband visible and near-infrared spectrum. The OPV device supported on these optimized nanogrooved substrates yields a 14% increase in short-circuit current over the corresponding flat device, highlighting the potential of this large-scale light-harvesting strategy in the broader context of thin-film technologies.

## 1 Introduction

A key challenge for sustainable global development relies on the possibility of exploiting clean and renewable energy sources. Renewable energy production based on solar energy conversion in photovoltaics (PVs) has attracted a great deal of both scientific and technological attention. The amount of solar energy reaching the Earth in just 1 h (4.3 × 10^20^ J) would indeed be sufficient to fulfill the energy consumption on the planet in a whole year (4.1 × 10^20^ J)^1^ provided the conversion efficiency of current solar energy conversion technologies is substantially improved.

In recent years, organic photovoltaics (OPVs) have emerged as a promising candidate owing to their potential merit of low-cost energy conversion^2^ and optional mechanical flexibility.^3−5^ It opens up a range of novel and unconventional applications, e.g., integrating smart building components,^6−8^ products, and electronic devices. Despite the substantial progress in the last years,^9^ the power conversion efficiency (PCE) of thin-film OPVs is still lower than their inorganic counterparts, and device performance always toggles between sufficient light absorption and good charge carrier extraction.^10^ Good light absorption would require a micrometer-scale thick active layer, restricting the collection of minority carriers.^11^ On the other hand, a thin layer of the organic absorber is preferable to collect efficiently the photo-generated carriers and beneficial against photon-induced degradation of the organic absorber. However, this limits the photon-absorption efficiency, which in turn reduces the overall efficiency of solar cells. A considerable portion of the incoming light is lost due to the reflection from the electrodes as well as due to incomplete absorption by the semi-transparent nature of the organic solar cells.

To suppress light reflection losses, various light management strategies have been proposed, e.g., the addition of anti-reflective thin-film coating.^12^ However, anti-reflective layers act over a limited wavelength range and suffer from delamination issues, which hinder mechanical stability in the long term.^13,14^ The use of metallic nanostructures supporting localized plasmon resonances has been extensively studied to exploit the resonant light scattering amplification and/or the near-field enhancement;^15−18^ however, the ohmic losses limit the efficiency of these strategies. In a complementary approach, anti-reflection and light trapping functionalities can be achieved by introducing subwavelength dielectric nanostructures on the transparent window, which supports the active OPV layer.^19−21^ A substantial increase of the photon absorption in thin-film photovoltaics has been demonstrated by functionalizing transparent or semiconductor substrates with anisotropic high-aspect aspect ratio nanostructures fabricated via several means, e.g., lithographic techniques,^22,23^ etching processes through nanofabricated masks,^24^ self-organized methods expoliting ion-beams^25−27^ bottom-up approach using colloidal spheres^28^, or via engineering more complex reconfigurable nanopatterns as recently demonstrated.^28−30^ Alternatively, flat-optics configurations have been recently devised in the case of ultrathin few-layer transition metal dichalcogenide semiconductors to promote strong in-plane light confinement via resonant excitation of guided photonic anomalies.^31,32^ In the case of thin-film devices (thickness in the range of 100–300 nm) supported on periodically textured surfaces endowed with subwavelength structures (SWSs) light coupling into the active thin film can be improved by significantly reducing reflection losses, mimicking the moth-eye effect: high-aspect--ratio SWSs fetch gradual changes in refractive index from the value of air to that of the substrate, leading anti-reflective functionality in the broadband spectral range.^33−37^ In a complementary way, dielectric nanostructures with a lateral size comparable to or larger than the incident wavelength behave as Mie resonators, strongly promote photon scattering and absorption in the active layer, and thus enhance the short-circuit current (*J*_SC_) in the thin-film PV device.

Here, we demonstrate the capability to improve the photoconversion efficiency in thin-film OPV devices via large-scale surface nanopatterning of the transparent window promoting broadband light trapping into the active thin layer. We investigate periodically surface-textured substrates, such as one-dimensional (1D) periodic gratings of different pitch (*P*) and groove height (*H*), to explore anti-reflection and Mie scattering effects for photon harvesting in organic thin-film devices. A cost-effective approach based on laser interference lithography (LIL), combined with RIE, provides homogeneous nanofabrication of 1D nanogratings over macroscopic areas (cm^2^) at the surface of transparent silica substrate and enables controlled tailoring of the nanostructure shape and periodicity. These templates are conformally coated with a thin organic absorber layer (PTB7: PCBM), investigating their characteristic optical response via transmission and reflection optical spectroscopy, to identify the optimum light trapping condition. A strong amplification of the optical absorption, as high as 19%, has been detected over a broadband visible and near-infrared spectral range, thanks to an optimized nanograting morphology (height 280 nm, period 300 nm). The nanopatterned thin-film device hosted on this engineered template finally showed an amplified photoconversion figure with a 14% increase of the short-circuit current with respect to the reference flat device. These results demonstrate the potential of the large-scale nanogratings for broadband photon harvesting and conversion in thin-film organic devices.

## 2 Materials and Methods

### 2.1 Building OPV Device on Silica Nanogratings

The fabrication of a thin-film OPV device on 1D silica gratings was achieved by an original variant of LIL technique combined with RIE process followed by controlled deposition of a series of thin films. A brief overview of the fabrication steps is schematically illustrated in Figure S1 (see Supplementary Information), and the details of each step are summarized below:

#### 2.1.1 Polymer Template Fabrication

To achieve 1D nanograting arrays over a large area of silica substrates, we developed an original variant of LIL for the fabrication of a large-area patterned template on a transparent substrate. In the first step, silica substrates were cleaned in an ultrasonic bath with acetone followed by isopropyl alcohol (Figure S1a). Then, a thin layer of a positive tone photoresist film (AZ 701 MIR diluted in the AZ-EBR solvent in a ratio of 2:1) was spin-coated on top of the cleaned silica substrate at a speed of about 2000 rpm for 60 s, immediately followed by a soft baking at 100 °C for 300 s on a hot plate (Figure S1b). At this point, we employed a 40 mW diode-laser source at wavelength λ of 406 nm (single longitudinal mode) aligned to our customized Lloyd’s mirror interference setup to impress an optical interference fringe pattern into the polymeric thin film (Figure S1c). To this end, the sample was post-baked at 100 °C for 300 s and immersed into developer (AZ 726 MIF) to achieve laterally separated polymer stripes over a large area of silica substrate (Figure S1d). Before laser exposure, we coupled a neutral density filter (NDF) of high optical density (OD: 4.00) behind the photoresist-coated substrate via a refractive index matching fluid (MF) (Cargille immersion liquid, OHGL nD). In this way, we eliminated the unwanted reflections originated from the back interface of the transparent substrate, which can form interference fringes when superimposed with incoming light, deteriorating the desired pattern in the photoresist.

#### 2.1.2 Stencil Masks for RIE Etching

The polymer stripe pattern was then decorated with Au overhanging structures (nominal thickness of 60 nm) from both sides by grazing incidence deposition of Au at an angle of 80° with respect to the surface normal (Figure S1e). Au was evaporated at a rate of 6 nm/min using a resistively heated alumina crucible in a high vacuum chamber (∼10^–7^ mbar range). A calibrated quartz crystal microbalance was employed to monitor the deposition rate and thickness of the deposited metal. At this point, aluminum (Al) was deposited on the prepared template at normal incidence in vacuum conditions (∼10^–6^ mbar range) at the rate of 9 nm/min using a resistively heated boron nitride crucible (Figure S1f). After Al deposition, the polymeric mask was lifted off via sonication within few minutes using an acetone solution. Thus, we obtained highly ordered and large-area Al nanostripes (Figure S1g) on silica. Au overhanging structures protect the sidewalls of the polymeric stripes from the metal coating during Al deposition, which facilitates efficient lift-off. After lift-off, the Al stripe mask was employed to selectively etch the silica substrates via a fluorine radical plasma produced in a RIE system (Oxford instruments plasma lab system 100). As a gas reactant, tetrafluoromethane (CF_4_) was used. During etching, the radio frequency (RF) power was set to 100 Watt, and the chamber pressure was kept at ∼30 mTorr. The lower etching rate of Al with respect to silica leads to the etching of well-ordered 1D nanograting structures on silica (Figure S1h). Details of the employed RIE parameters are summed up in Table S1.

#### 2.1.3 Developing OPV Device on Silica Nanogratings

**Figure 1:**
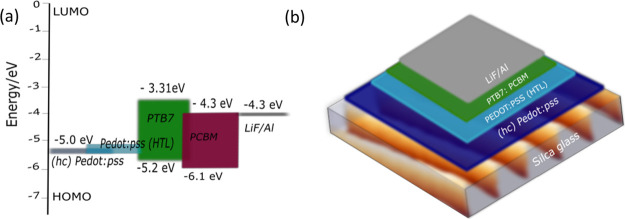
(a) Energy level diagram of the organic layers employed in the OPV device. (b) Device architecture of the nanogrooved organic solar cell.

In order to study the light trapping effect in OPV device, we employed poly[[4,8-bis[(2-ethylhexyl)oxy]benzo[1,2-b:4,5-b′]dithiophene-2,6-diyl][3-fluoro-2-[(2-ethylhexyl)carbonyl]thieno[3,4-*b*]thiophenediyl]], (PTB7, 1-materials inc.) as a donor part. As an acceptor, phenyl C_61_ butyric acid methyl ester (PC_61_BM, Ossila) is used. Both materials are blended with a final concentration of 25 mgmL^–1^. We mixed 10 mg of PTB7 to 15 mg of PC_61_BM in a chlorobenzene/1,8-diiodooctane mixed solvent (97:3 vol %) and left it over a magnetic stirrer at 75 °C overnight. Both flat and nanograting silica templates are prepared with a gold busbar. A highly conductive solution of poly (3, 4-ethylenedioxythiophene) doped with poly (styrene-sulfonic acid) (PEDOT: PSS, Baytron/Clevios PH 1000) and modified with 5% dimethyl sulfoxide, DMSO, is coated on top. Accordingly, the (PEDOT: PSS (hc)/5% DMSO) acts as a bottom conductive and transparent electrode. A hole transport layer (PEDOT: PSS, Baytron/Clevios PH 500) is spin-cast at 2000 rpm for 2 s and 4000 rpm for 45 s, then baked at 120 °C for 10 min. Then the active absorber layer (PTB7:PC_61_BM) is spun on top at 1500 rpm for 15 s and annealed at 80 °C for another 10 min. Substrates are directly transferred to the nitrogen-filled glove box for the deposition of the top contact. Finally, in thermal evaporation, a pressure of 2 × 10^–6^ mbar is achieved for the deposition of 0.7 nm LiF/100 nm Al to complete the device assembly, as presented in Figure 1b.

### 2.2 Measuring the Photovoltaic Properties

Devices were connected to a Keithley 236 source meter and placed under Steuernagel 575 solar simulator with 100 mW cm^–2^ intensity to record the current density–voltage characteristics under AM1.5 solar irradiation. The photocurrent response and the external quantum efficiency (EQE) were recorded using a home-build setup with a Xenon-lamp, an ACTON Spectra Pro150 monochromator, and an EG&G 7260 DSP Lock-in amplifier. Typical monochromatic illumination intensities of 5–10 μW were used for the measurements.

### 2.3 Atomic Force Microscopy

The nanostructured patterned morphology has been investigated ex situ utilizing atomic force microscopy (AFM) operating in tapping mode (Nanomagnetics ezAFM). High-aspect-ratio Si tips of nominal tip radius 10 nm (probe PPP-XYNCSTR-50) were employed. Typically, the images of resolution 1024 × 1024 pixels were acquired by keeping the fast axis of the scanner along the wave vector of the 1D grating structure. The morphological characteristics, e.g., period, height, and width of the grating topography, were extracted from the AFM images using the open-source software WSxM (version 5.0 Develop 6.51).^38^

### 2.4 Far-Field Optical Spectroscopy

To characterize the anti-reflective and scattering properties of the nanostructured samples, we have performed far-field spectroscopic measurements in transmission and reflection configuration. An optical beam from a white light source (halogen and deuterium lamp DH-2000-BAL, Mikropak) was coupled to a linear polarizer (Glan-Thompson) through an optical fiber with a core diameter of 600 μm, which finally impinged on the sample. Then, the light transmitted or reflected by the samples has been fiber coupled to the PC-controlled high-resolution solid-state spectrometer supplied by Ocean Optics (HR4000) operating in the spectral range of 300–1100 nm. Total transmission and reflection spectra were collected employing an integrating sphere. The schematics of the optical measurement configurations are displayed in Figure S2.

### 2.5 Absolute Absorption Measurements

In order to quantify the absorption property of the organic layers, we placed the sample inside the integrating sphere setup which is illustrated in Figure S2c. At first, we acquired a reference dark spectrum with the light source switched off to count the detector noise level. Secondly, we recorded the reference blank spectrum to capture the optical background when the sample is still inside the sphere, but shifted out from the direct light beam path, indirectly illuminated by light reflected on the sphere walls (*T*_Indirect_). Finally, we recorded another spectrum with the sample mounted in the incident light path, i.e., in a direct illumination condition (*T*_Direct_). In this way, the light trapped in the sample by total internal reflection and escaping from the side can also be obtained. The normalized integrated signal was calculated by *T* = (*T*_Direct_ – Dark)/(*T*_Indirect_ – Dark), and the total absorption was obtained by *A* = (1 – *T*). For the measurements, the input light source (halogen and deuterium lamp DH-2000-BAL, Mikropak), optical fibers and the solid-state detector (Ocean Optics, HR4000) are the same used in the transmission and reflection measurements mentioned in the previous section.

## 3 Results and Discussions

### 3.1 Nanogrooved Organic Photovoltaic Devices

Thin-film organic photovoltaic devices have been developed by selecting an heterostack based on PEDOT: PSS, PTB7 and PCBM, endowed with optimal band alignment properties for photoconversion, as shown by their energy level diagram shown in Figure 1a.^39^ To promote efficient optical coupling, the OPV devices have been grown onto transparent silica window, while a LiF/Al thin film has been exploited as electrode.

In this study, we devise a novel optical configuration of this thin-film device based on nanogrooved silica templates as a transparent window (Figure 1b). In this configuration, the incident light can be effectively coupled to the OPV device via photonic light trapping strategies.

### 3.2 Nanofabrication of Substrates and Characterization

**Figure 2:**
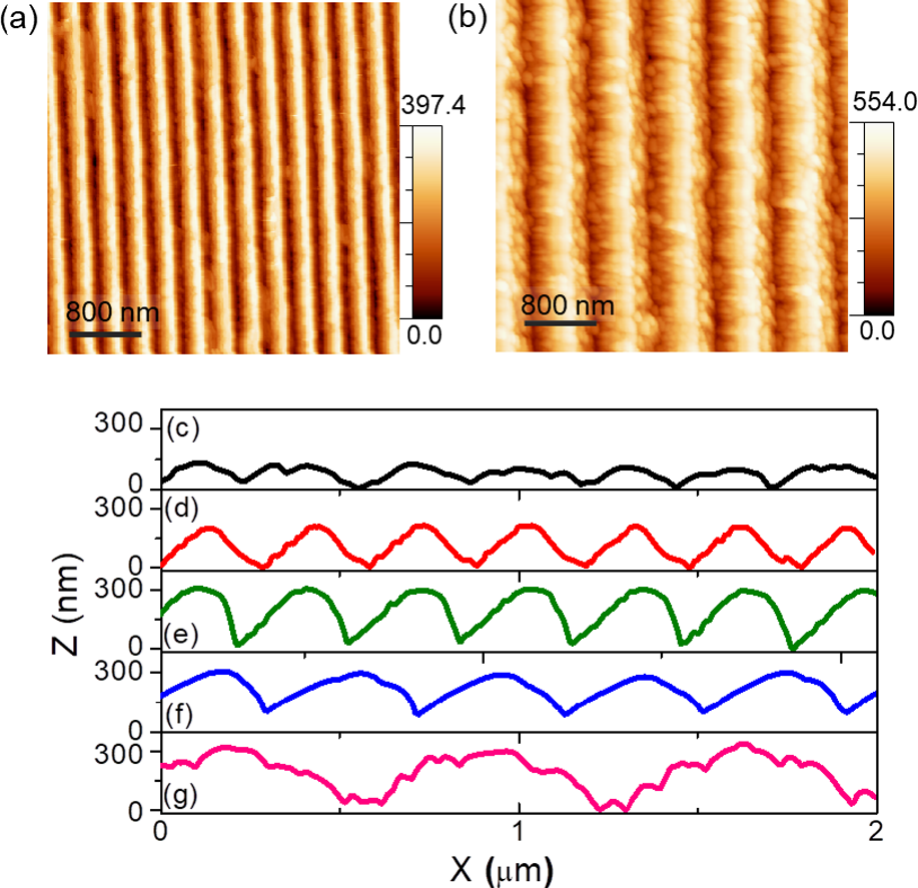
Panels (a) and (b) display representative AFM topography acquired on samples 3 and 5, respectively. In the lower panel, line profiles corresponding to the AFM images of Figure S4 are shown. Grating period (*P*) and amplitude (*H*) of the corresponding sample are (c) Sample 1; *P* = 293 ± 6 nm and *H* = 140 ± 30 nm (black line); (d) Sample 2; *P* = 295 ± 6 nm and *H* = 213 ± 10 nm (red line); € Sample 3; *P* = 294 ± 8 nm and *H* = 280 ± 10 nm (green line); (f) Sample 4; *P* = 402 ± 6 nm and *H* = 290 ± 7 nm (blue line); and (g) Sample 5; *P* = 706 ± 3 nm and *H* = 300 ± 5 nm (magenta line), respectively.

The controlled fabrication of anisotropic 1D grating structures by RIE over large-area (cm^2^) silica substrates has been achieved through an original variant of LIL. The details of the LIL process are described in the Materials and Methods section. This cost-effective nanofabrication approach has enabled us to produce highly ordered nanogratings on photoresists with tailored periodicity, which remain highly stable with time and are extended uniformly over cm^2^ area of flat and transparent silica substrates. We achieve polymer templates of different periods, *P* = 293 ± 8 nm, 400 ± 8 nm, and 710 nm ± 12 nm, in order to tailor light manipulation in the OPV devices (see AFM micrographs in Figure S3 in the Supplementary Material). These period values are found in good agreement with the theoretical prediction, *P* = λ/2 sin θ , which leads to *P* = 287, 406, and 780 nm under coherent laser illumination at the wavelength λ = 406 nm for incidence angle (θ) = 45° ± 1°, 30° ± 1°, and 15° ± 1°, respectively. The details of the chosen LIL parameters, e.g., laser power, duration of laser exposure, development time etc., are summarized in Table S2 of the Supplementary Material. We present a photograph of one of the LIL fabricated samples in Figure S3a to illustrate the large-area homogeneity of the highly ordered nanopatterned polymeric thin film. Optical diffraction fringes observed in the film area ∼ 2 × 2 cm^2^ validate the presence of nanopatterns on a macroscopic portion of the substrate surface. The polymer templates were then employed as a stencil for the deposition of the required Al mask during the RIE etching of the Silica substrate to form subwavelength uniaxial gratings (see the Materials and Methods section for experimental details). To increase light coupling into the OPV cell, the morphology of the nanogratings has been optimized. The height *H* of the nanostructures has been modified by controlling RIE exposure time, while their periodicity *P* is controlled by changing the illumination angle during LIL interference lithography. In our experiments, we either increased ripple amplitude at fixed period *P* ≈ 295 nm (for samples 1, 2, and 3, the height, respectively, reads *H* ≈ 140, 213, and 280 nm), or we increased the period at fixed amplitude *H* ≈ 290 nm (for samples 3, 4, and 5, the period, respectively, reads *P* ≈ 294, 402, and 706 nm). Two representative AFM topographies of small period silica template (Sample 3) and large period template (Sample 5) are shown in Figure 2a,b allow characterizing the representative patterns with highly ordered anisotropic lattices of different height (*H*) and period (*P*). The full set of AFM topographies corresponding to samples 1–5 is available in Figure S4 of the Supplementary Material.

The statistical analysis of the AFM images in Figure S4 results in *H* values of 140 ± 30 nm for Sample 1 (Figure S4a), 213 ± 10 nm for Sample 2 (Figure S4b), and 280 ± 10 nm for Sample 3 (Figure S4c) while the average period remains constant around 294 ± 6 nm for all samples. On the other hand, Figure S4c–e highlights grating with a constant average grating height of 290 ± 10 nm while varying period to *P* = 294 ± 8 nm (Sample 3), 402 ± 6 nm (Sample 4) and 706 ± 3 nm (Sample 5). To achieve these grating structures, the chosen RIE time, the description of the active agents, and the other processing parameters are tabularized in Table S2 of the Supplementary Material. For a better visualization of the height and the periodicity of the grating structures plotted in the AFM images of Figure S4, we have extracted the surface profiles shown in the lower panel of Figure 2.

### 3.3 Optical Characterization of the Nanostructured Substrates

**Figure 3:**
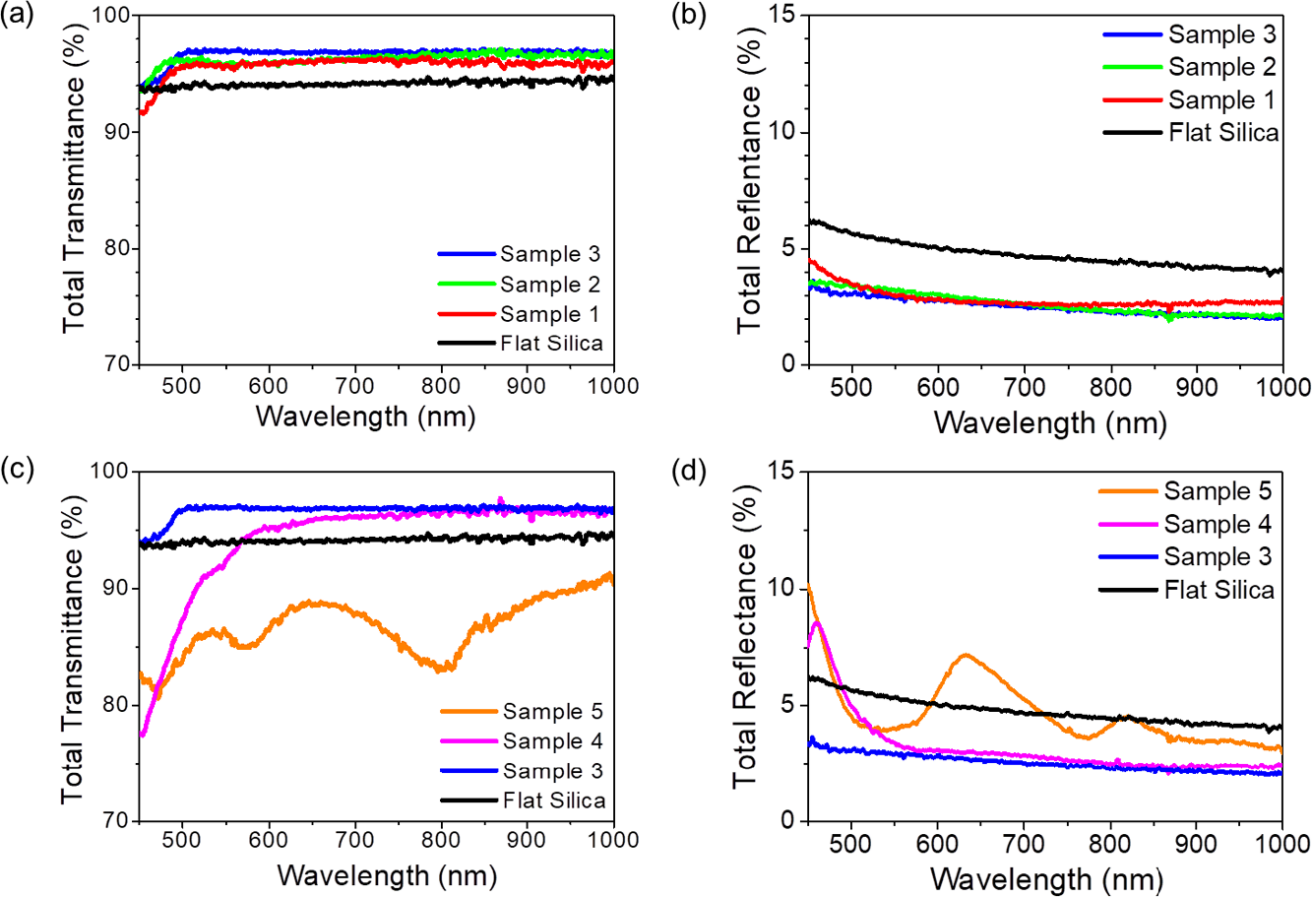
Observation of optical behaviors measured from both the nanostructured and flat silica. (a) and (c) show the total integrated transmittance spectra, while (b) and (d) summarize total integrated reflectance spectra for different samples. (a) and (b) correspond to Sample 1, Sample 2, and Sample 3, whereas (b) and (d) characterize Sample 3, Sample 4, and Sample 5, respectively. All the spectra have been measured by an integrating sphere employing unpolarized light.

To evaluate the light scattering properties of the templates, we fully characterize both the nanostructured and the flat silica templates by integrating transmission and reflection measurements in far-field optical spectroscopy. All the spectra were detected by exploiting an integrating sphere setup that can be configured either in transmission or in reflection (see Supporting Information, Figure S2 for further details). Since in both these configurations, the light component eventually waveguided parallel to the sample surface (*W*) is not coupled to the detector, in Figure 3 we show both total transmission spectra (*T*, Figure 3a,c) and total reflection spectra (*R*, Figure 3b,d) of the flat and nanogrooved silica templates. In this way, it would be possible to calculate the in-plane waveguided fraction as *W* = 100%–*T*–*R*. Figure 3a,b shows the spectra of nanogrooved templates endowed with the same grating periodicity but of different pattern amplitude, while Figure 3c,d refers to templates of different periodicity and same pattern amplitude. In Figure 3a the spectrum corresponding to flat silica (black trace) shows a total integrated transmittance value around 94 ± 1% over the broad range of visible to NIR wavelengths. By introducing periodic grating structures on silica, we detected a prominent increase in the optical transmission at the wavelength range from 500 nm and above. For instance, an increase of transmittance around 1.7% over the spectral range of 450–1000 nm is achieved for grating structures of height 140 nm and period 293 nm (sample 1, red trace in Figure 3a). The occurrence of the anti-reflective effect over such a broadband range of wavelengths is attributed to the “moth-eye” effect.^24^ This effect occurs when the light wavefront traverses the region between two dissimilar media of different refractive indices, which are coupled by a transition layer featuring a gradual transition of the refractive index (index-grading effect). In our approach, such smooth transition in refractive index between dissimilar media (air and silica) is obtained by introducing a dense array of nanostructures at the silica interface, characterized by subdiffractive periodicity and high aspect ratio. In order to ensure a smoother transition of the refractive index, we vary the height of the nanostructures by keeping fixed the grating periodicity at about 294 nm. Intuitively, we expect that the nanostructures characterized by higher aspect ratio (*h/w*) favor a smoother refractive index transition while moving from air to silica, promoting the anti-reflection effect. The green line of Figure 3a corresponds to the spectra of Sample 2 (nanostructures height increased to 213 nm): we detect an absolute improvement of the transmittance up to 2.3%. A further increase of the nanostructure height to 280 nm (Sample 3, blue line in Figure 3a) reveals a saturation in transmittance value around 2.4%.

In order to further confirm the anti-reflective behavior of the nanogratings and to rule out the possible role of diffuse reflection, we have also measured the integrated reflection spectra (direct plus diffuse components) from all the templates. For this purpose, we employed the same apparatus used in transmission measurement but in reflection configuration (see the schematic in Figure S2b). As shown in Figure 3b, for flat silica (black trace in Figure 3b), the average reflectance reads 6.3%, and for the grating structures of amplitude 140 nm (Sample 1, red trace in Figure 3b), we observe a reduction in reflectance around 1.7% within the broadband spectral range from visible to NIR wavelengths. A further drop in reflectance value around 2.6% is observed when the taller grating structures of amplitude 280 nm (Sample 3, blue trace in Figure 3b) is considered. The reduction percentages in the reflectance incorporated by the SWS grating structures are found in good agreement with the increase of their corresponding transmittance values, as described earlier.

It is to be noted that the transmittance spectra displayed in Figure 3a, starting from low corrugation amplitude (Sample 1) to high amplitude (samples 2 and 3), show a drop in transmittance below wavelength 500 nm, which is not due to the absorption (negligible in silica for such wavelengths), but rather to light trapping and waveguiding within the silica slab. In fact according to simple considerations based on the grating equation, the drop in transmittance at low wavelengths originates from diffraction and scattering from the corrugated structures, once light wavelengths are comparable or smaller than the grating period.

In the final optimization step we further explored the possibility of optimizing photon harvesting by keeping the height of the nanostructures fixed at the optimal value of 280 nm, which provides maximum transmittance, and by increasing the periodicity of the nanogratings to 402 ± 6 nm (sample 4) and 706 ± 3 nm (Sample 5). Indeed if the period of the nanogratings increases, a larger spectral portion of the incident light will get scattered and diffracted into propagating modes, which can strongly perturb the angular distribution of light flow, favoring off-specular light scattering at large angles, which in turn will maximize light coupling to the active layer of the solar cell. In Figure 3c, we plot the total integrated transmittance spectra corresponding to Sample 4 (magenta trace) and Sample 5 (orange trace) together with Sample 3 (blue trace) and a reference flat silica (black trace). Compared to flat silica, the spectrum acquired from nanograting structures of period 402 nm (Sample 4) shows a significant drop of 6.8% in transmittance in the small wavelength range (averaged from 450 to 567 nm) and an increase of 1.7% in the long-wavelength range (averaged from 567 to 1000 nm). Notably, the end part of the spectrum in the NIR overlaps with that of small period gratings (Sample 3, period 294 nm) for which antireflection moth-eye effects are dominant given the subdiffractive size of the nanofeatures. In addition, the spectra of Sample 4 shows a shallow transmission dip at about λ_P_ = 546 nm, which can be described in the context of diffractive anomalies. The latter are generally observed near the evanescent transition and are either described as Rayleigh anomalies (RA) or as a guided-mode anomaly (GMA),^40−42^ depending if light propagation takes place tangent to the external interface or trapped and waveguided by total internal reflection. In both cases, the following equation holds,
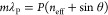
1where *m* is the diffraction order, λ_P_, denotes the peak wavelength, *P* is the grating period, θ represents the incidence angle of light (chosen to be slightly off-normal to avoid artifacts in the integrating sphere measurements) and *n*_eff_ corresponds to either the refractive index of the substrate (∼1.46 for SiO_2_) for the RA, or the effective index of the guided-mode anomaly excited along with the array for the GMA.^41,42^ We thus calculated the value of *n*_eff_ using eq 1, which reads 1.2 for the first order mode *m* = 1. The value is significantly lower than the refractive index of SiO_2_ substrate, but higher than that of air, and indicates that the propagation of the diffracted light is confined in the silica substrate (and not in the air interface). The scattering and diffraction anomalies become more pronounced when the grating period increases to 706 nm (Sample 5, orange trace in Figure 3c), as revealed by the 12% drop in the transmittance averaged over the whole spectral range. The corresponding transmittance spectrum also displays two significant dips at λ_P_1__ = 571 nm and λ_P_2__ = 803 nm. Considering eq 1, those transitions correspond respectively to second order (*m* = 2) RA in the effective medium with *n* = 1.47 and the first order (*m* = 1) Rayleigh anomaly in the effective medium with *n* = 0.99 corresponding to the air interface. The transmittance behavior shown by both samples 4 and 5 is complemented by the total integrated reflectance measurements shown in Figure 3d, which evidence an overall drop in the average reflectance for the respective grating samples compared to the flat reference substrate.

### 3.4 Light Trapping in Organic Thin-Film Absorbers

**Figure 4:**
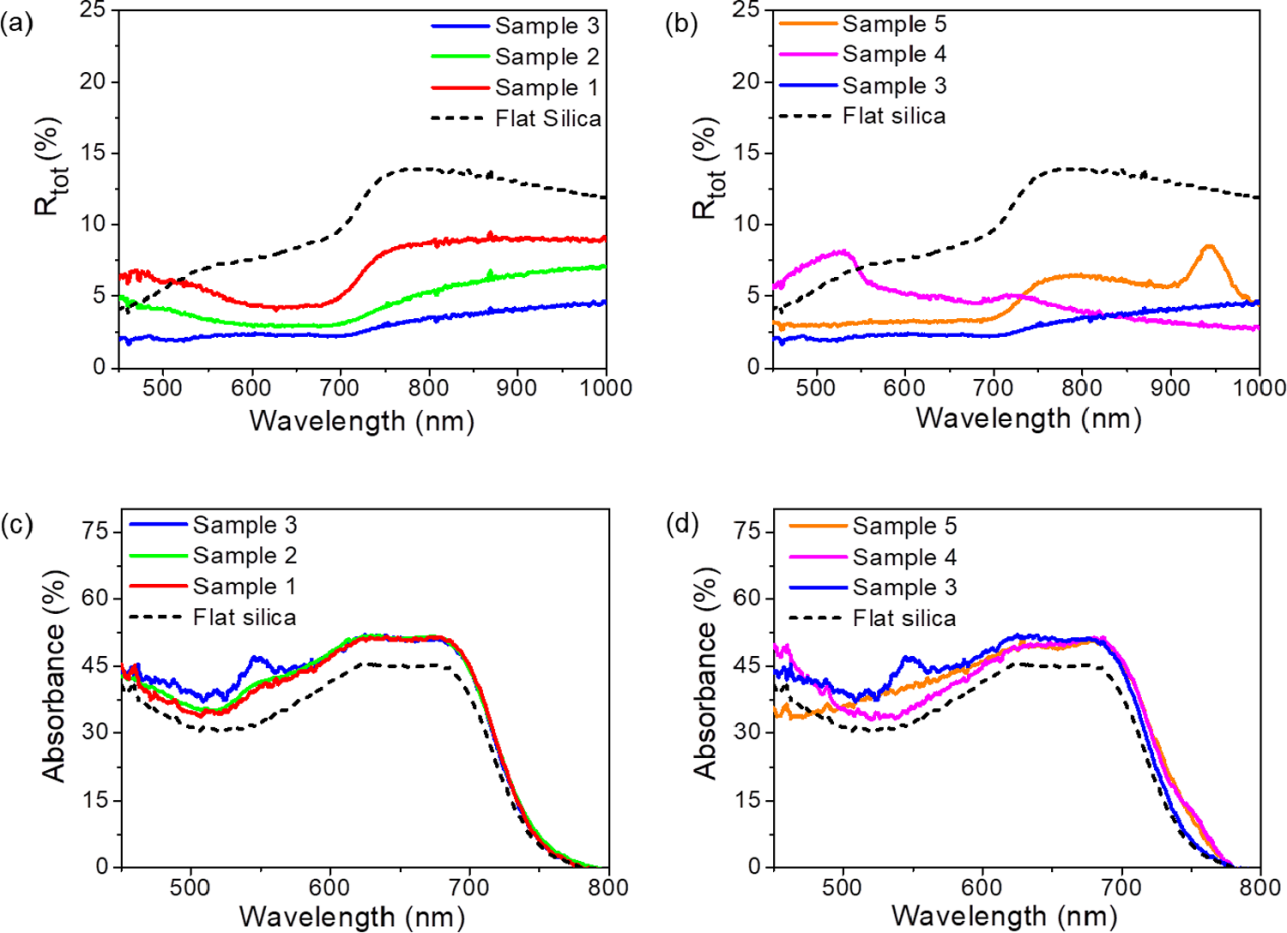
(a) Reflection measurements of the organic absorber layer grown on patterned silica and flat silica substrates using an integrating sphere in an open configuration. The spectra represent samples of average period 295 nm but of different heights 140 nm (Sample 1, red trace), 213 nm (Sample 2, green trace), and 280 nm (Sample 3, blue trace). On the other hand, (b) corresponds to the spectra acquired on the nanogratings of average height 286 nm but of different periods 295 nm (Sample 3, blue trace), 402 nm (Sample 4, magenta trace), and 706 nm (Sample 5, orange trace). (c) and (d) represent the absorption measurements of the organic absorber layer grown on flat silica substrates and patterned silica samples 1-2-3 and samples 3-4-5, respectively using an integrating sphere in closed configuration.

In order to increase the photon absorption and carrier generation in thin layers of organic photovoltaic semiconductors, we grew them on the corrugated dielectric interfaces which feature the light trapping strategies described above. In the following, we study the optical characterization of a 100-nm-thick organic absorber layer (PTB7: PCBM) grown by spin-coating both on the flat and on the nanograting silica substrates. This represents the basic element of a realistic device architecture with the subwavelength structures (SWSs) placed on the front side of the solar cell. Figure 4a,b shows the total reflectance acquired with the integrating sphere setup in an open configuration (the measurement schematic is shown in Figure S2b). The data reveal that the textured solar cells grown on Sample 3 achieve a substantial reduction in total reflectivity (specular plus diffuse) over the whole spectral range, not only compared to the flat reference but also with respect to the other patterned samples, confirming previous observations made on the bare silica substrates. More quantitatively, in Figure 4c,d, we plot the absorption spectra acquired from the flat as well as from the grating samples using an integrating sphere setup in a closed configuration, i.e., when the sample is mounted inside the sphere and the sphere collects total reflected, total transmitted and also waveguided light (Figure S2c).

In Table 1, we summarize the quantitative analysis by integrating the absorption spectra over the relevant wavelength range. Figure 4c compares absorption in corrugated cells with variable amplitude and fixed period (*P* = 294 nm). Notably, the maximum value of normalized absorption (36.1%) integrated over the whole spectral range from 450 to 800 nm is achieved with the nanograting structures of height 280 nm (Sample 3). This should be compared to the spectrally averaged absorption of 30.3% measured on flat silica. On the other hand, Figure 4d compares absorption in corrugated cells with constant amplitude *H* around 280 nm and variable period *P*. Sample 3 with the smallest period *P* = 294 nm performs substantially better compared to the flat reference and also compared to the other samples with a larger period. In this case, however, we observe that for samples with a larger period (samples 4 and 5), there is a crossover at longer wavelengths, approximately above 700 till 800 nm, compared to the flat reference and sample 3. The latter can be attributed to an increase in the effective optical path length of photons in the active organic layer due to the scattering from the larger structures. The best relative gain in absorption, around 19% for Sample 3 relative to the flat reference, indicates that for this morphology (*P* = 294 nm, *H* = 280 nm), there is an optimal tradeoff between moth-eye anti-reflection effect from the small-scale structures and light trapping due to scattering from the larger structures.

**Table 1:** Summary of the Absorption Measurements Shown in Figure 4c,d Acquired from the Organic Layers Supported on Nanostructured and Flat Silica Substrate Integrated over the Spectral Wavelengths 450–800 nm

details of support substrates	*P* (nm)	*H* (nm)	normalized absorption (%)	absolute increase in normalized absorption (%)	relative increase in normalized absorption (%)
flat silica			30.3		
sample 1	293	140	35.1	4.7	15.5
sample 2	295	213	35.4	5.1	16.8
sample 3	294	280	36.1	5.7	19.0
sample 4	402	290	35.2	4.9	16.0
sample 5	706	300	35.1	4.7	15.0

Also, it is worth noticing that the rippled samples 1, 2, and 3 show a further increase of absorption compared to the flat film within a relatively narrow band at around 545 nm, with sample 3 exhibiting a more pronounced effect compared to samples 1 and 2.

### 3.5 Simulation of the Light Trapping Properties of Thin-Film Absorbers

To investigate the origin of the observed features and deepen our understanding of the mechanisms presiding over the absorption increase from rippled configurations of the organic layer, we performed a numerical simulation of the optical experiments of samples 1, 2, and 3 with the period around 295 nm and variable amplitude. A full-wave two-dimensional scattering model based on port-formalism was implemented in a commercial tool (Comsol Multiphysics 6.0). For the profile of the silica substrate, we mimicked the AFM topography of Figure 2 using a smoothed polygon curve with geometrical parameters as in Table 1, and the organic layer configuration was assumed to be conformal to the silica substrate, with a constant thickness of 100 nm. For the optical constants, we took the complex refractive index of PTB7:PC61BM reported in ref (43).

**Figure 5:**
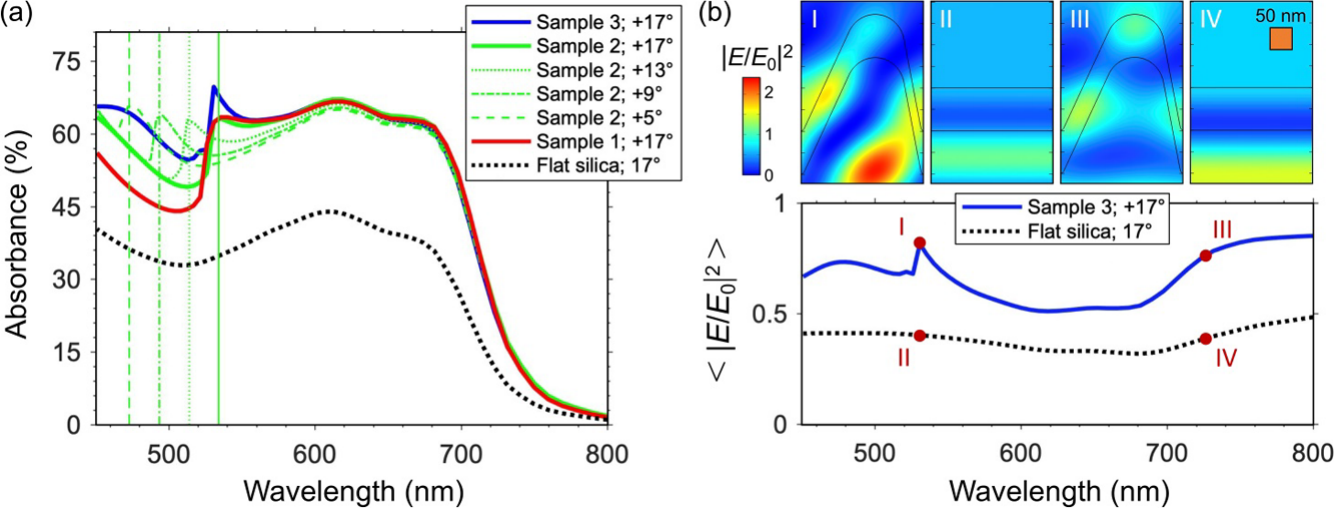
(a) Simulated absorption of the organic layer grown on flat silica substrate (black dotted curve) and patterned silica samples 1, 2, and 3 (red, green, and blue curves, respectively) under illumination from the substrate at 17° angle of incidence. Green dotted, dash-dot, and dashed curves also show the simulated absorption of sample 2 for 13°, 9°, and 5° angles of incidence, respectively. Vertical green lines highlight the spectral position of RA in the silica substrate for the considered set of angles. (b) Simulated field enhancement averaged on the organic layer for the flat film (black dotted curve) and for sample 3 (blue curve) at 17° angle of incidence. Top insets show the field enhancement patterns corresponding to the samples and spectral positions marked by red dots in the main panel.

Figure 5a shows the calculated absorption spectra of the samples, evaluated as *A* = 1 – *T*_tot_ – *R*_tot_, with *T*_tot_ and *R*_tot_ the total transmittance and reflectance of the structure under illumination from the glass substrate. We found good agreement with the experimental results of Figure 4c for 17° angle of incidence. Note that the numerical analysis confirms both the broadband increase of absorption for the rippled organic layers compared to the flat layer, and the further absorption increase over a narrow band centered at around 540 nm for samples 1, 2, and 3 (compare solid curves with black dotted curve in Figure 5a), with sample 3 (blue curve) showing the strongest effect compared to samples 2 and 3 (red and green solid curves). The numerical study also enabled us to identify the origin of these effects. In particular, a decrease in the angle of incidence results into a blue shift of the narrow band peak (compare green curves in Figure 5a), and the spectral position of this peak well matches the Rayleigh anomaly of the grating in the silica substrate,^32^ highlighted by vertical green lines in Figure 5a.

This interpretation is further confirmed by inspecting the near fields achieved in the organic layer. Figure 5b shows the electric field enhancement spectrum |*E*/*E*_0_|^2^ (*E*_0_ is the electric field of the incident wave) averaged over the organic layer for the flat film configuration (black dotted curve) and for the rippled film configuration corresponding to sample 3 (blue solid curve). Note that the sample 3 exhibits a higher field enhancement on the whole spectral range under consideration, with a peak precisely sitting at around 540 nm, that corresponds to a complex spatial distribution (inset I in Figure 5b) typical of a grazing diffraction order, i.e., of a Rayleigh anomaly (also compare with the field distribution in the flat film, shown in inset II of Figure 5b). For longer wavelengths, where the grating only supports fundamental reflection and transmission orders, the near-field pattern exhibits a very different configuration, characterized by hot spots located at the crests and valleys of the rippled profile (inset III in Figure 5b). Note that the size of these hot spots is comparable to the thickness of the polymer layer. This indicates that the broadband increase of absorption in samples 1, 2 and 3 is precisely due to the capability of the active layer to concentrate light at the nanoscale. Such a possibility is enabled by both the rippled configuration (cf. inset IV in Figure 5b) showing the poor concentration of light in the active layer for the flat configuration and the relatively high permittivity of the polymer, which is of the order of 4 in the considered wavelength range,^43^ i.e., significantly higher than that of the silica substrate. In a similar configuration of the rippled profile but for ultrathin MoS_2_ nanolayers as active medium,^32^ such an effective light concentration was not allowed being the active layer too thin (∼4 nm). Therefore, the broadband increase of light absorption in MoS_2_ was due to a different mechanism: the interaction of nonresonant scattered light, guided into the bulk substrate, with the 2D nanogratings.

Finally, we highlight that the absorption increase retrieved by the numerical analysis overestimates the measured one by a factor of almost 3 (cf. Figures 4c and 5a). This can be attributed to a departure of the polymer layer from the conformal configuration (assumed in the simulations), and indicates a significant margin of improvement of light concentration capability in corrugated films of PTB7: PCBM with optimized deposition conditions.

### 3.6 Organic Photovoltaic Devices

**Figure 6:**
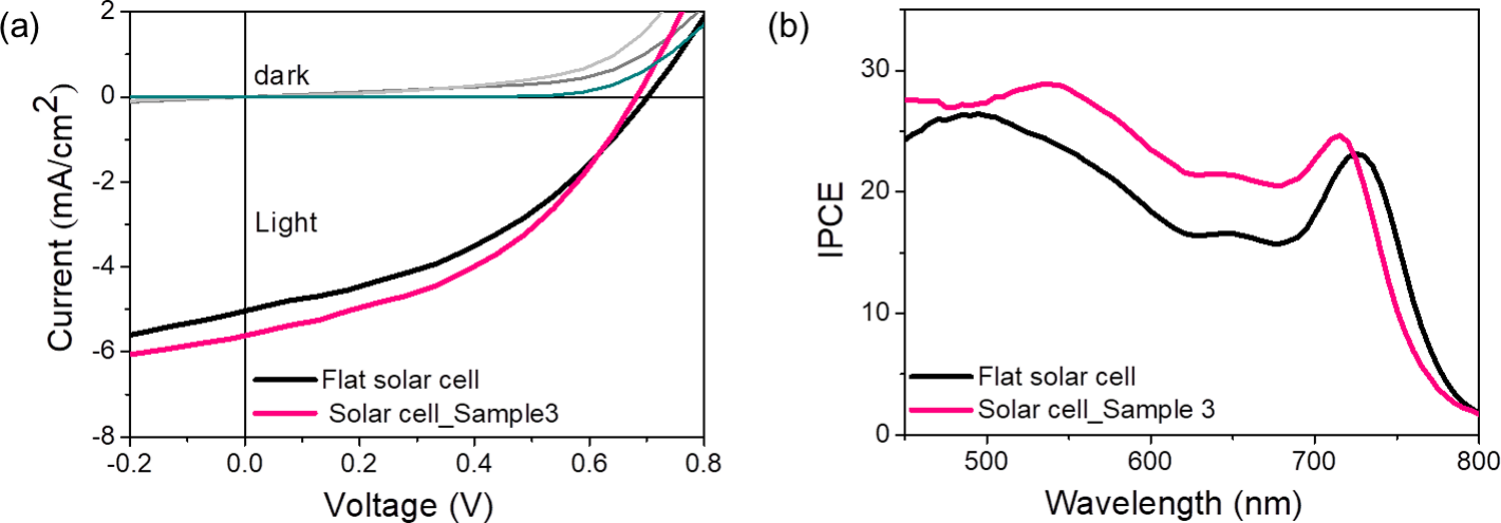
(a) Current–voltage characteristic and (b) the external quantum efficiency, EQE, of the organic solar cells fabricated onto the nanostructured template (pink line) and onto the flat (black line) silica substrate.

To elucidate the impact of the nanogrooved templates on the electrical performance of the absorber layer, we built the prototype solar cell device using the organic layer on sample 3 (*P* = 294 nm and *H* = 280 nm) as well as on flat silica by applying PTB7: PCBM as an active absorber layer. The details of the fabrication steps are summarized in the Materials and Methods section. The energy level diagram of the layers employed in the device fabrication and the device architecture scheme are shown in Figure 1a,b.^39^ The measured current–voltage characteristics, *J*–*V* curves, and the EQE of the photovoltaic cells are presented in Figure 6a,b respectively. On the other hand, the quantitative parameters as extracted from the *J*–*V* curves are summarized in Table 2 (data is determined from the average of four devices). The current density, *J*_SC_ values in Table 2 are estimated by integrating the EQE spectra to minimize the error from the active area calculations. A detailed look into the results indicates device parameters of 5.72 mA/cm^2^ for *J*_SC_, 704 mV for *V*_OC_, 45.39% (FF), and 2.23% of PCE, respectively for a photovoltaic cell built on the nanogroove pattern compared to *J*_SC_ of 5.05 mA/cm^2^, *V*_OC_ of 683 mV, 41.73% FF, and 1.82% PCE for the flat photovoltaic cell. As a significant outcome, there is a 14% relative increase in the *J*_SC_ value is observed for the device built on the nanogroove pattern compared to its flat counterpart. The improvement in the photocurrent value attributed to the overall better light-in coupling to the absorber layer of 18% due to moth-eye-antireflective effect, scattering, and light trapping phenomena as revealed from the spectrally resolved EQE data of Figure 6a,b. The 4% mismatch between the *J*_SC_ value and photon harvesting effect is attributed to the recombination losses of photoexcited carriers before collection. These losses are possibly related to the increase in surface roughness of the interface and confirmed by the reduction in the *V*_OC_ value of the patterned solar cell device, which measures 683 mV compared to 704 mV in the flat device.

**Table 2:** Summary of the Electrical Behavior Shown in Figure 4c,d Collected from Nanograting and Flat Solar Cells^a^

	*J*_SC_ [mA/cm^2^]	*V*_oc_ (mV)	FF (%)	Eff	*R*_s_ (Ω)	*R*_sh_ (Ω)
flat solar cell	5.058	704	41.73	1.82	162.5	2330.80
patterned solar cell_sample3	5.724	683	45.39	2.23	121	3495.32

a Values are determined from four devices, and *J*_SC_* values are corrected according to the EQE spectra

## 4 Conclusions

We investigated photon harvesting properties in organic photovoltaic devices exploiting large-area nanograting substrates, which act as supports for the active organic absorber layer. The nanograting structures extending over cm^2^ areas of the substrate have been fabricated by a cost-effective nanofabrication approach based on LIL, combined with controlled metal evaporation and RIE. Overall, we observe a 19% relative increase in absorption probability in the thin layer of organic absorber PCBM: PTB7 compared to the flat reference for the optimal grating morphology. Also, the organic solar cell device grown on the same grating structures shows a remarkable improvement of the short-circuit current density *J*_SC_, in the order of 14%. Our results thus indicate that the periodically corrugated silica templates so far described effectively promote light-harvesting in thin-film organic solar cell devices. The high-aspect-ratio nanograting structures substantially enhance light transmission in the VIS–NIR spectral range due to anti-reflective moth-eye effect, while light trapping and scattering are dominant in the blueshifted part of the spectrum at smaller wavelengths. Efficient manipulation of incident light flow and coupling to the active absorbing layer following a scalable large-area approach is of interest also for a broader range of solar harvesting applications involving thin-film absorbers.

## Supporting Information Available

Schematic of the fabrication procedures, optical measurement schemes, RIE parameters for fabricating silica nanogratings of different height and periodicity, LIL parameters for fabricating polymer templates of different periodicity, AFM images of the polymer templates (PDF)

## Abbreviations

OPV: organic photovoltaic
1D: one-dimensional
PVs: photovoltaics
SWSs: subwavelength structures
LIL: laser interference lithography
RIE: reactive ion etching
NDF: neutral density filter
MF: matching fluid
RF: radio frequency
EQE: external quantum efficiency
