# Supplementary material for: Broadband Photon Harvesting in Organic Photovoltaic Devices Induced by Large-Area Nanogrooved Templates
Source: ACS Appl Nano Mater. 2023 Apr 3;6(7):6230–40. doi: 10.1021/acsanm.3c00553 (PMC10112484; doi:10.1021/acsanm.3c00553)
Supplement: Supplementary file 1 — an3c00553_si_001.pdf [file an3c00553_si_001.pdf]

Supporting Information

Broadband Photon Harvesting in Organic Photovoltaic Devices Induced by Large-area Nanogrooved Templates

Debasree Chowdhury[1] †, Shaimaa A. Mohamed[2,3,4,5], Giacomo Manzato[1], Beatrice Siri[1], Roberto Chittofrati[1], Maria Caterina Giordano[1,], Mohamed Hussein[2,6,7], Mohamed F.O. Hameed[2,4,-8,] Salah S.A. Obayya[2,9], , Philipp Stadler[5], Markus. C. Scharber[5], Giuseppe Della Valle[10], Francesco Buatier de Mongeot[1,*]*

[1]University of Genova, Department of Physics, Via Dodecaneso 33, 16146, Genova, Italy.

[2]Centre for Photonics and Smart Materials, Zewail City of Science, Technology and Innovation, October Gardens, 6th of October City, Giza 12578, Egypt.

[3]Centre for Nanotechnology, Zewail City of Science, Technology and Innovation, October Gardens, 6th of October City, Giza 12578, Egypt.

[4]Nanotechnology and Nanoelectronics Engineering Program, Zewail City of Science, Technology and Innovation, October Gardens, 6th of October City, Giza, 12578 Egypt.

[5]Linz Institute for Organic Solar Cell (LIOS), Physical Chemistry, Johannes Kepler University Linz, Altenbergerstr, 69, A-4040 Linz, Austria.

[6]Department of Physics, Faculty of Science, Ain Shams University, Abbassia 11566, Cairo, Egypt.

[7]Light Technology Institute, Karlsruhe Institute of Technology, Engesserstrasse 13, 76131 Karlsruhe, Germany

[8]Mathematics and Engineering Physics Department, Faculty of Engineering, University of Mansoura, Mansoura 35516, Egypt

[9]Department of Electronics and Communication Engineering, Faculty of Engineering, University of Mansoura, Mansoura, Mansoura 35516, Egypt

[10] Dipartimento di Fisica and IFN-CNR, Politecnico di Milano, Piazza Leonardo da Vinci, 32-20133 Milano, Italy

***Emails:** buatier@fisica.unige.it, giordano@fisica.unige.it

1. Fabrication of Silica nanogratings:

We achieve one-dimensional silica nanogratings using Laser Interference Lithography (LIL) combined with Reactive Ion Etching (RIE), the corresponding fabrication steps are schematicaly illustrated below:

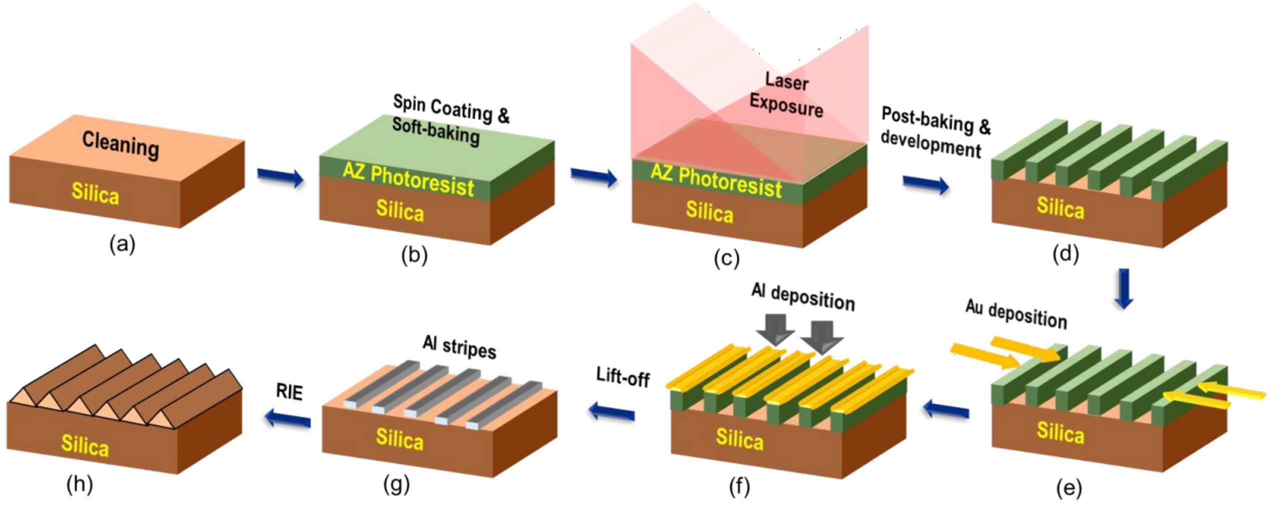

Figure S1. Schematic of the fabrication procedures for the grating nanostructures on Silica. (a) Cleaning of Silica substrate in an ultra-sonic bath with acetone followed by isopropyl alcohol. (b) Spin coating and soft baking of polymer photoresist resist AZMIR701 on the cleaned silica substrate. (c) Recording interference fringes into the resist layer using laser interference lithography (LIL). (d) Post-baking followed by development of the laser exposed photoresist surface to generate disconnected polymer stripes. Depositing (e) Au overhanging structures on resist stripes at grazing incidence 80° and (f) Al mask at normal incidence. (g) Removing polymer stripes through lift-off to obtain Al stripes on silica substrate. (h) Processing of these Al stripes through reactive ion etching (RIE) which results highly-ordered nanogratings on silica substrate.

2. Optical setup

To fully characterize the optical properties of the nanogrooved templates and of the OPV devices we performed integrated transmission and integrated reflection measurements by coupling an unpolarized white light beam to an integrating sphere setup. The two configuration employed for the integrated transmission and reflection measurements are shown in Figure S2 a, S2 b respectively. Both the setups are based onto an integrating sphere whose internal surface is homogeneously coated with a highly diffusive Spectral on film. Under this condition the detector, that is fiber coupled to the sphere out of the principal beam, effectively collects either the total transmittance (Figure S2a) or the total reflectance (Figure S2b) corresponding to the principal beam and the diffused optical intensity. However in both the configurations of Figure S2 a, b the light intensity waveguided parallel to the sample surface is not coupled to the detector. In order to collect also the waveguided light we developed an alternative configuration as shown in Figure S2 c: in this latter case the sample is placed into the integrating sphere and the sum of the total reflectance, the total transmittance and the waveguided light can be effectively coupled to the detector, allowing the

direct measurement of the optical absorbance into the sample.

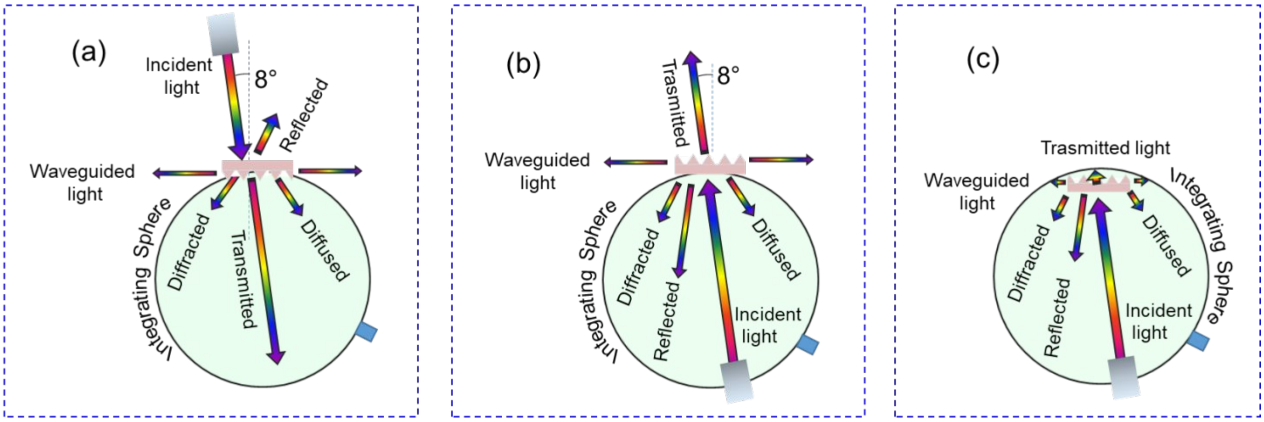

Figure S2. (a,b) Sketches of the optical setups employed for the integrated transmission and integrated reflection measurements, respectively; in both the cases the light component waveguided parallel to the sample is not coupled to the detector. (c) A follow-up configuration optical setup that allows direct measurement of the sample absorbance by placing the sample into the integrating sphere. In this case the whole optical intensity (Transmittance +Reflectance +Waveguided) is coupled to the detector.

3. Table S1:

Series of silica nanogratings of different hight and periodicity are fabicated following the RIE parameters described below:

Sample	Aluminium thickness (nm)	Gas reactant	Power (W)	Pressure (mtorr)	RIE time (min)
1	50	Tetrafluoromethane (CF_4)	100	30	14
2	65	Tetrafluoromethane (CF_4)	100	30	8
3	85	Tetrafluoromethane (CF_4)	100	30	10

4	90	Tetrafluoromethane (CF_4)	100	30	13
5	65	Tetrafluoromethane (CF_4)	100	30	11

4. Table S2:

To nanofabricate polymer templates of different periodicity, employed LIL parameters are described below:

**Sample**	**AZ MIR 701/ AZ EBR**	**Dilution time (min)**	**Laser Exposure time (sec)**	**Laser Power (mW)**	**θ w.r.t surface normal (degree)**	**Dose (mJ/cm^2)**	**Development time (sec)**
1, 2, 3	1:2	15	25	0.574	45	23	5
4	1:2	15	25	0.563	30	22	5
5	1:2	15	38	0.413	20	25	5

4. Tuning the periods of the polymer template:

We achieve polymer fringes of different periodicity (P) by simply chaning the laser illumination angle (θ) and the corresponding pattern morphologies are presented via the AFM micrographs below:

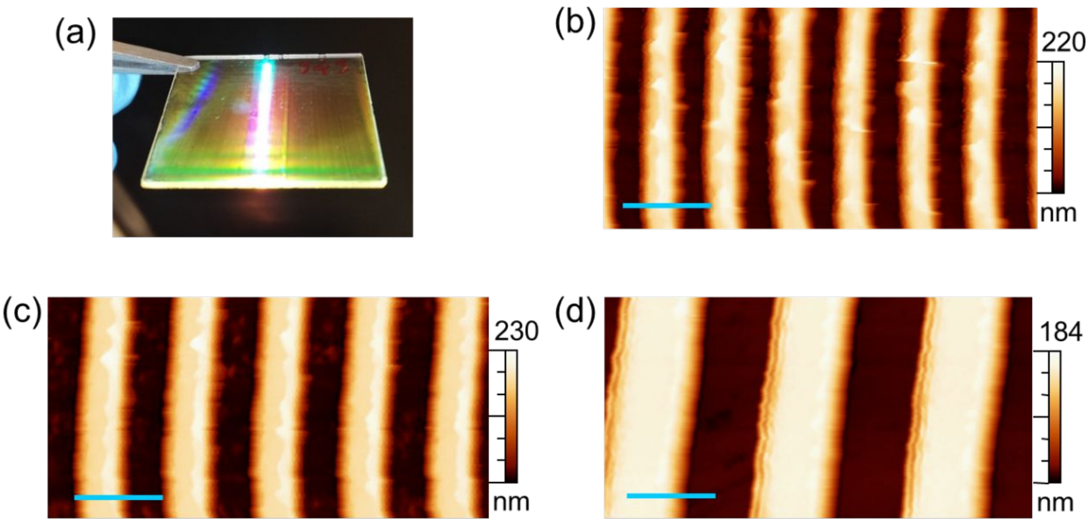

Figure S3. (a) shows photograph of 1D photoresist gratings supported on the large-area silica substrate. (b), (c) and (d) represents AFM images of the polymer patterns developed on silica substrates with periodicity (a) 295±6 nm, (b) 400±8 nm and (c) 710 nm±12nm; through LIL at laser illumination angles (θ) 45°, 30° and 15° respectively. Blue scale bar in each image corresponds to 400 nm.

The photoresist mask of Figure S3 is employed for deposition of Al stencil hardmask for Silica Reactive Ion Etching. After RIE etching through the Al etching mask (Step h of Figure S1) the silica templates is patterned with gratings of different amplitude and same period (panels S4 a, b, c correspond to samples 1, 2, 3 respectively), or to samples with similar amplitude and different period (panels S4 c, d, e correspond do Samples 3, 4, 5 respectively)

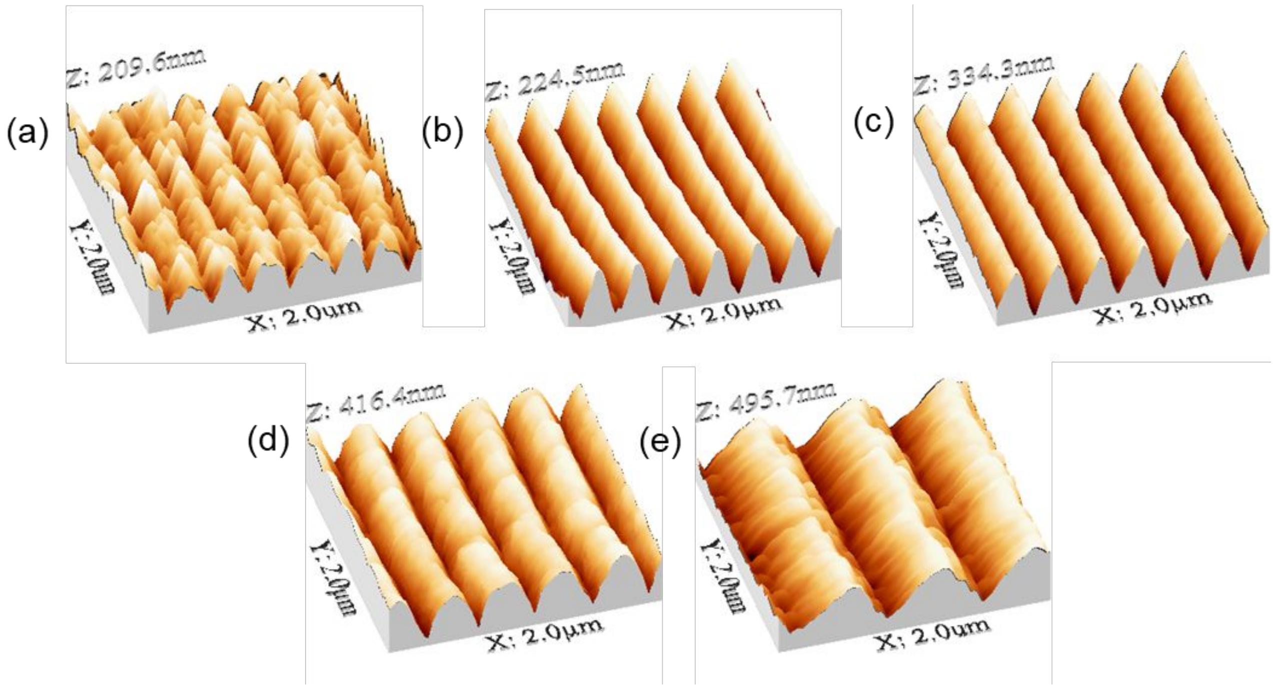

Figure S4. Panel (a-e) display AFM topography of the silica templates, acquired on Samples 1, 2, 3, 4 and 5 respectively. Grating period (P) and amplitude (H) of the corresponding sample are (a) Sample 1; P = 293±6 nm and H = 140±30 nm ; (b) Sample 2; P = 295± 6 nm and H = 213±10 nm ; (c) Sample 3; P = 294±8 nm and H = 280±10 nm ; (d) Sample 4; P = 402± 6 nm and H = 290± 7 nm ; and (e) Sample 5; P = 706± 3 nm and H = 300±5 nm respectively.
